# Avoiding Bleeding in the Modified Bentall Procedure

**DOI:** 10.1055/s-0041-1725120

**Published:** 2021-10-07

**Authors:** Uberto Bortolotti

**Affiliations:** 1 Division of Cardiac Surgery, University Hospital, Pisa, Italy

After more than 50 years from the original description, the modified Bentall procedure (MBP) still represents the gold-standard treatment for patients with aneurysms of the ascending aorta and with an aortic valve not amenable to repair. 1 The MBP implies several suture lines, all of which are at risk for postoperative bleeding, especially in the presence of fragile tissues, as in patients with aortic dissection or Marfan syndrome. Techniques providing accurate hemostasis are, therefore, a prerequisite for a successful outcome and reduction of postoperative morbidity. This is particularly true when the proximal suture is performed, which fixes the composite conduit in place; in fact, once the aortic cross clamp is released, any bleeding site from the posterior aspect of such suture is almost impossible to reach.

**Fig. 1:**
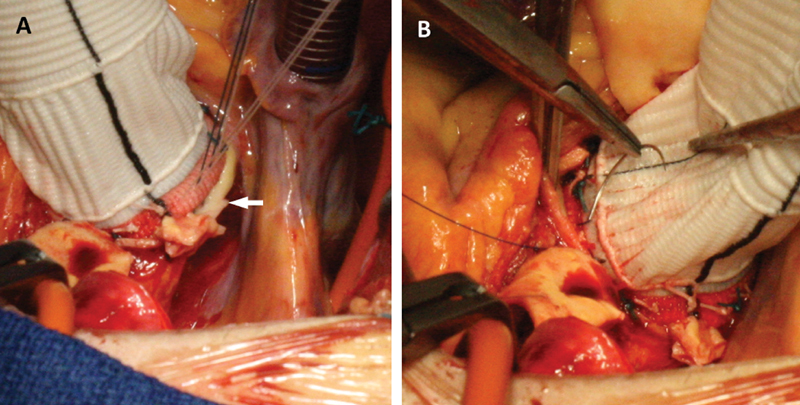
( **A** ) In the modified Bentall procedure, a rim of aortic wall is visible after the conduit is seated (arrow). ( **B** ) A stitch of 3/0 polypropylene is used to reinforce the proximal suture.

In the February 2020 issue of *AORTA* , Kasai et al 2 described another technique to avoid postoperative bleeding from the proximal anastomosis after the MBP. In our experience, we have used, since the early 1990's, the technique proposed by Copeland et al 3 in 1993, consisting of reinforcement of the proximal suture line with an additional suture. 3 In our initial experience, when using this modification of the MBP, we observed a significant reduction in postoperative bleeding, the need for blood transfusions, and chest reexplorations. 4 My colleagues and I have continued to routinely perform the MBP using this additional suture line with extreme satisfaction ( Fig. 1 ); it takes less than 5 minutes to be performed, it does not influence the length of ischemic time, and it does not affect either morbidity or mortality.

Kasai et al 2 have used the technique they describe using a home-made mechanical conduit. This is quite surprising given the large variety of currently available mechanical conduits, most of which include the Valsalva graft. Therefore, I feel that the technique they propose may probably find more application when utilizing biological conduits for the MBP, which have to be constructed in the operating room. In my opinion, when performing the MBP with mechanical conduits, use of commercially available devices with a reinforcing not time-consuming proximal suture line, appears sounder.
